# Are There Ethnic Differences in Hand Eczema? A Review

**DOI:** 10.3390/jcm12062232

**Published:** 2023-03-14

**Authors:** Eleanor Shu Xian Chai, Hong Liang Tey, Ziying Vanessa Lim

**Affiliations:** 1 Duke-NUS Medical School, Singapore 169857, Singapore; eleanorchai@u.duke.nus.edu; 2 National Skin Centre, Singapore 308205, Singapore; drvanessalim@gmail.com; 3 Lee Kong Chian School of Medicine, Nanyang Technological University, Singapore 308232, Singapore; 4 Yong Loo Lin School of Medicine, National University of Singapore, Singapore 117597, Singapore

**Keywords:** dermatitis, contact, racial, culture, geography

## Abstract

Hand eczema is a common disease with economic and social ramifications. This study undertakes a review of certain existing literature to provide insight into contributory factors which may result in the varying prevalence and severity of hand eczema among different ethnic groups, particularly to identify modifiable risk factors, as well as to ascertain knowledge gaps for future research direction. The existing literature suggests that factors including (a) genes, (b) differing skin physiology, (c) cultural practices, (d) dietary habits and associated food preparation, (e) climate, (f) predominant occupations, (g) socioeconomic factors, and (h) dissimilar laws and regulations may account for the disparity in the risk of hand eczema among different ethnicities. Given that endogenous factors cannot be avoided, but certain exogenous aspects can be modified, especially as the environment plays an important role in hand eczema flares, it is helpful from a practical perspective to focus on addressing the modifiable risk factors. These factors pertain to unique cultural practices, customs, and food preparation methods. Healthcare professionals should be well-acquainted with such factors to tailor the treatment approach for patients of different ethnicities accordingly because, with globalization, physicians face increasingly diverse patient populations such that cultural customs no longer remain limited to particular geographic regions.

## 1. Introduction

Hand eczema is an inflammatory skin disorder predominantly involving the dorsal and palmar aspects of the hands. The pathogenesis is multifactorial, contributed by a combination of endogenous factors, such as genetic predisposition of atopic dermatitis and exogenous elements, such as irritants or allergens [1,2]. These features alter the structure and composition of the stratum corneum to disrupt the skin barrier, thereby leading to the development of hand eczema [1].

Hand eczema, a potentially chronic condition, is common globally, with an estimated lifetime prevalence of around 14.5% in the general population [3]. There is a significant economic and social impact of hand eczema [4] due to the direct costs of treatment, indirect costs, including absenteeism and job losses, and psychological effects on patients’ quality of life [5,6]. Hence, early diagnosis and prompt intervention are vital to reducing the likelihood of progression to chronic hand eczema [7], which may result in resistance to topical treatment [8]. Chronicity of hand eczema is defined by at least three months’ duration or relapses at least twice a year despite adequate treatment adherence [9]. Based on the etiology, hand eczema can be broadly classified into (a) atopic hand dermatitis, (b) irritant contact dermatitis, (c) allergic contact dermatitis, (d) hybrid hand eczema, and (e) idiopathic. Categorization of the morphology includes (a) vesicular or dyshidrotic hand eczema (pompholyx), (b) hyperkeratotic hand eczema, (c) chronic fingertip dermatitis, (d) nummular hand eczema, and (e) dry, fissured hand eczema [10].

Several studies have examined the association between occupational exposure and hand eczema, but few have examined the disparities of hand eczema incidence and severity along ethnic lines. Although not widely formally studied, there is suggestion that the prevalence of hand eczema may vary between different ethnicities. Therefore, the aim of this review is to provide insight into possible contributory factors that may result in the varying risk of hand eczema in different ethnic groups, in particular, to identify modifiable risk factors for healthcare professionals to consider when formulating therapeutic approaches, as well as to identify knowledge gaps for future research direction.

## 2. Materials and Methods

A review was performed broadly in reference to the Preferred Reporting Items for Systematic reviews and Meta-Analyses extension for Scoping Reviews (“PRISMA-ScR”), with adaptations as appropriate for the current context.

### 2.1. Search Strategy

The literature available on the online databases MEDLINE via PubMed, EMBASE, Scopus, Cochrane Library, *New England Journal of Medicine*, and *The Lancet*, published from the relevant inception date to 10 February 2023 in English, were searched using a systematic search strategy. The search terms used on MEDLINE via PubMed were (“hand eczema” OR “hand dermatitis” OR “hand dermatoses”), (dermatitis[MeSH Terms] AND “ethnic”), (dermatitis[MeSH Terms] AND “race”), (dermatitis[MeSH Terms] AND “nickel”), (dermatitis[MeSH Terms] AND “aromatherapy”), (eczema[MeSH Terms] AND “ethnic”), (eczema[MeSH Terms] AND “race”), (eczema[MeSH Terms] AND “nickel”), (eczema[MeSH Terms] AND “aromatherapy”), (“dermatoses” AND “cultural”), (“contact allergy” AND food), as well as ((“hand eczema” OR “atopic dermatitis”) AND “vitamin D”), and the search terms on the other databases mentioned were (“hand dermatitis” AND “ethnic”), (“hand dermatitis” AND “race”), (“hand dermatitis” AND “cultural”), (“hand eczema” AND “ethnic”), (“hand eczema” AND “race”), (“hand eczema” AND “cultural”), and (“dermatoses” AND “cultural”). The studies were then assessed based on title and abstract for relevance to hand eczema and association with ethnicities.

### 2.2. Selection of Articles: Inclusion and Exclusion Criteria

To be included in the review, papers needed to measure or focus on one or more specific factors or measures that may result in an increased risk of dermatoses (including hand eczema) in different ethnic groups. Quantitative and qualitative studies, including systematic reviews, meta-analyses, randomized controlled trials, prospective and retrospective cohort studies, and case-control studies that compared the prevalence or severity of dermatitis were considered in order to take into account elements that may contribute to hand eczema in different ethnicities.

Studies were excluded if they did not fit into the conceptual framework of the study, where full text was not available or accessible, or if not published in English.

### 2.3. Data Extraction and Analysis

All relevant papers were identified through two stages. At the first stage, articles were screened for eligibility against the review criteria by reading the title and abstract from the search results, where available. At the second stage, the full text of each of the articles identified from the first stage was then read to determine its relevance. The full text of eligible articles was obtained for data extraction. Key data extracted from the eligible papers included (a) the basic study characteristics, (b) the type of dermatosis examined (in particular, whether it pertains to hand eczema), and (c) the underlying conceptual approach specified or implied, including how the component(s) analyzed affects the prevalence or severity of such dermatosis in an ethnic group.

## 3. Results

**Figure 1:**
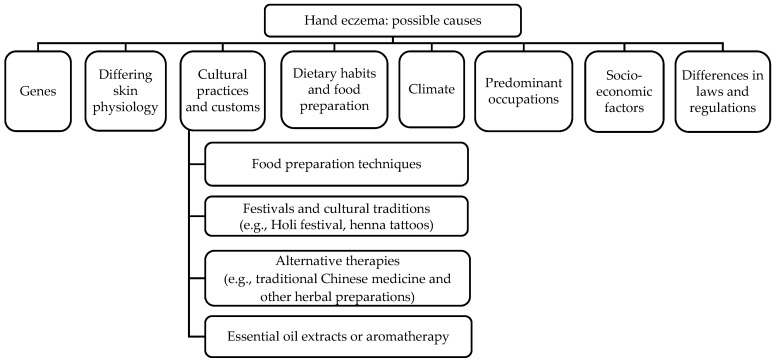
Possible causes of hand eczema across different ethnicities.

In this review, the titles and abstracts of 6355 publications were screened, of which 171 publications were identified for full-text review and from which 79 publications were selected. The disparity in hand eczema prevalence and severity may be attributed to a combination of exogenous and endogenous reasons, detailed below (Figure 1).

### 3.1. Genetic Factors

Several studies have proposed that certain genes may increase the risk of or offer protection against hand eczema. The heterozygous loss-of-function mutation in the filaggrin gene, in combination with atopy, negatively affects the disease course of hand eczema. The tumor necrosis factor (TNF) α-238A allele has a protective effect against irritant hand dermatitis, while the TNFα-308A allele is a risk factor for irritant hand dermatitis. The homozygous and heterozygous status of the late cornified envelope genes LCE3B and LCE3C has significant associations with chronic allergic contact hand dermatitis. The SPINK5 gene has a significant association with non-atopic hand dermatitis, and the interleukin 1A gene is considered to have protective effects with regard to irritant hand dermatitis [11,12].

In particular, the mutation(s) in the filaggrin gene may increase the risk of certain sub-type(s) of hand eczema due to the contribution toward a disrupted epidermal barrier, greater loss of hydration, inflammation, and exposure to environmental allergens [13]. However, these associations are contentious as Lerbaek et al. [14] and Carlsen et al. [15] noted no association between the filaggrin null alleles and hand eczema, but the former acknowledged that this could be due to insufficient statistical power, and the latter recognized that such mutation(s) could, nevertheless, have an impact on certain sub-types of hand eczema, such as in the context of irritant hand dermatitis. Moreover, more recent literature cites that filaggrin mutation(s) are associated with early onset, persistence, and/or an adverse disease course of hand eczema [11,16,17]. Specifically, Molin et al. [18] propose that such mutation may contribute to the development and maintenance of a particular sub-type of chronic hand eczema characterized by the combination of allergic and irritant contact dermatitis.

Filaggrin mutations vary in terms of prevalence among different ethnicities [19], which may explain why different ethnicities have different incidences and severity of hand eczema. “Evolutionary pressures” has been cited to explain the higher rate of filaggrin mutations in Europeans and relatively lower rates in non-European ethnic groups [20], and such mutations appear to not be as prevalent in African American patients [21], although certain papers were in the context of atopic dermatitis and not hand eczema specifically. Additional investigations are required to determine if the other genetic mutations discussed are more prevalent in any particular ethnic group.

### 3.2. Differing Skin Physiology

Differing skin physiology among the various ethnic groups may represent different sensitivity levels to chemicals, affecting the prevalence and severity of hand eczema. Asian skin may be more sensitive to exogenous chemicals due to an intrinsically thinner stratum corneum and higher density of eccrine glands [22]. Despite having the lowest ceremide levels, darker skin types may have a stronger barrier against chemical or mechanical insults [22] due to the presence of a more compact stratum corneum [23]. Muizzuddina et al. [24] investigated the differences in skin barrier function and structural elements of the stratum corneum of African Americans, Caucasians, and East Asians who lived in the same geographical region to reduce the impact of environmental exposure differences and found that East Asian skin is more sensitive than Caucasian skin, which is, in turn, more reactive than the skin of African Americans, because East Asian skin, and to some extent Caucasian skin, had low maturation and relatively weaker skin barrier.

Separately, differing skin pigmentation of various ethnic groups may result in varying skin sensitivity. Several studies have found that individuals with darker skin tend to have lower levels of vitamin D, possibly partly because increased pigmentation may reduce vitamin D production, and vitamin D, in turn, may protect against the development of atopic dermatitis [25,26]. This is because vitamin D may assist in reducing inflammation of the skin via mechanisms such as (a) modulation of structural proteins in the skin, which regulate glycoseramides required for the lipid barrier and thereby keeping the skin moisturized, (b) reducing the release of pro-inflammatory cytokines and inhibiting the release of IgE, and (c) inhibiting monocyte production and dendritic cell activity [27]. Although there are contradictory reports (such as a cohort study that suggested no significant association between vitamin D level and risk of atopic dermatitis [28] and a study that found no major correlation between vitamin D levels, atopic dermatitis severity, and serum LL-37 levels [29]), several of the existing literature propose vitamin D deficiency is a prominent risk factor of atopic dermatitis [30], and some clinical trials indicate that vitamin D supplementation may reduce disease severity [31,32,33,34]. Larger scale in-depth investigations may be required to confirm the association [33,35].

### 3.3. Cultural Practices and Customs Leading to Exposure to Chemicals

Some ethnic groups may have increased exposure to certain chemicals due to cultural practices and customs, which could heighten the risk of hand eczema incidence and severity.

This could arise due to the food preparation of cuisine unique to ethnic groups, detailed in Section 3.4.

Secondly, certain festivals and cultural traditions involve the use of chemicals that increase the risk of hand eczema. For example, the Holi festival in India involves the preparation and widespread scattering of colored substances, resulting in cases of irritant or allergic contact hand dermatitis in festival goers [36,37,38]. Henna is used as hair dyes and temporary decorative tattoos in certain cultural celebrations, such as weddings, the exposure to which may result in contact dermatitis [39,40] and IgE-mediated hypersensitivity reactions [36], especially in hairdressers and henna artists [41].

Alternative homeopathic therapies adopted by some ethnic groups may also heighten the risk of dermatological conditions. While certain alternative treatments are purported to address dermatological lesions with varying efficacy, the topical application or consumption of traditional Chinese medicine and other herbal preparations (made of, for example, tea tree oil, camomile (Anthemis nobilis), Rhus toxicodendron, French marigold (Tagetes patula), or cumin oil) may increase the risk of allergic contact dermatitis [39,42]. An illustration is a case of herbal patch preparations of mugwort (Artemisia vulgaris) causing a rash on the patient’s hands and other contact areas [43].

The common use of essential oil extracts or aromatherapy in various parts of the world (including China, India [36,39], and Australia [44]) has been cited by various studies to potentially increase the likelihood of hand eczema [45]. In particular, hand dermatitis was noted to be common among aromatherapists [39]. Based on a study involving 14 essential oils (which excludes, among others, lavender oil), the five most common essential oils that result in positive patch testing appear to be, in decreasing order, ylang-ylang, lemongrass, jasmine absolute, sandalwood, and clove oils [46], although more recent literature noted that neroli oil and narcissus absolute increasingly resulted in positive patch testing as well [47]. Cases of sensitization to lavender oil have been reported in aromatherapists and massage therapists, but lavender oil has not been included in certain patch testing investigations because, among other reasons, it appears that standardized test preparations of oxidized lavender oil are presently not easily commercially available [47]. Contact dermatitis arising from aromatherapy could possibly be due to direct and airborne exposure [48,49,50].

### 3.4. Dietary Habits and Food Preparation

Dietary habits and associated food preparation methods are plausible contributing factors. First, there could be variances in terms of food preparation leading to distinctive exposure risks. For instance, “capsaicin hand dermatitis”, a form of irritant contact dermatitis, was described in the Hispanic population due to the prevalence of handling cayenne and jalapeno peppers barehanded as condiments in Hispanic dishes [51,52]. Furthermore, pizza makers may present with hand eczema, believed to be due to contact with ammonium persulphate (present in flour) and diallyl disulphide (found in garlic, a common pizza ingredient) during food preparation [53].

Second, this could be due to the different types of food intake. While there is, at present, no clear direction in associations between hand eczema and diet [54], some studies suggest a positive association. In particular, one such study reported patients with vesicular hand eczema experiencing recurrences when they re-introduced particular food groups after completing an elimination diet, and another study described patients with vesicular hand eczema improving after following a prescribed diet [55]. In addition, Sharma et al. [56] noted the efficacy of a low-nickel diet in improving and/or reducing recurrences of hand eczema in nickel-sensitive individuals. Although such dermatological effects may have arisen due to an individual’s allergy profile, dietary intake may nevertheless be pertinent to consider because certain communities may potentially consume foods that contain comparatively higher nickel levels. For example, the Indian diet includes more plant-based foods that may contain relatively more nickel compared to the more animal-based Western diet. [57].

The lines regarding differing cuisine and food preparation techniques may be blurring due to increasing globalization and cross-border migration as individuals may be exposed to cuisine from other ethnicities, but it could still be useful to further determine how dietary habits and the associated food preparation could result in differing prevalence and severity of hand eczema, to be better placed to propose suitable preventive and treatment measures.

### 3.5. Climate

The climate, based on different geographical locations of ethnic groups, could be an element that affects the development and severity of hand eczema. Climatic conditions such as temperature, humidity, and ultraviolet radiation have been observed to affect the incidence and severity of dermatological conditions.

Higher latitudes and low temperatures are associated with a higher incidence of atopic dermatitis, whereas high temperature and humidity appear to be protective [20]. The severity of atopic dermatitis worsens with increasing latitude, while higher temperature reduces the severity, and ultraviolet light exposure (which, among other things, stimulates pre-vitamin D synthesis) reduces the severity of atopic dermatitis [25]. On the other hand, a large-scale, prospective, longitudinal cohort study determined that in the long term, higher temperatures, humidity, and more sun exposure are associated with poorly controlled eczema instead [58]. Therefore, the impact of a particular climatic condition on dermatological conditions does not appear so clear cut, possibly because distinct sub-types of eczema can be affected by climatic conditions differently, there is a complex interplay of various climatic conditions concurrently and/or the relationship between a climatic condition, and dermatological conditions may not be a dichotomy but a spectrum (i.e., rather than a climatic condition having a positive or negative effect only, there could be an optimal range or threshold instead, beyond which the effect of such climatic condition is reversed). For example, although humidity is considered to promote skin moisture and, thus, be protective (as detailed in the next paragraph), a study also purports the opposite effect that “warm and humid weather also promotes the evaporation of water on the skin surface, which may further exacerbate skin dryness, a characteristic clinical feature of eczema” [58].

In the context of hand eczema, a study suggests humidity may help to maintain “basal sweating responses” and, therefore, prevent hand eczema [59]. In addition, another study noted that “low temperature and low relative humidity tended to be risk factors” with respect to irritant hand dermatitis [60]. However, it could be said that no studies have sufficiently investigated this topic, thereby drawing no conclusions about the causality between climate and the prevalence of hand eczema [61]. Thus, more definitive investigations into the association of climatic conditions with hand eczema incidence and severity may be required.

### 3.6. Predominant Occupations in Various Ethnic Communities Affecting Exposure Risk

The nature of work predominant or present in different ethnic communities could also result in varying occupation exposures. Examples include hairdressers and artists that apply henna, who are at risk of hand and forearm dermatitis [41] and the prevalence of hand dermatitis among aromatherapists [39]. Tannery workers working in tanneries located in Indonesia are exposed to chemicals resulting in a heightened risk of contact dermatitis, in particular on their hands, wrists, and forearms [62]. A study highlighted the prevalence of hand dermatitis in two occupations that are popular among women in Nigeria: (a) dressmakers have a heightened risk of hand eczema because of their exposure to nickel found in scissors, pins, and needles; and (b) orange fruit sellers because of exposure to the chemicals from peeling oranges [63].

Another instance is the existence of occupational hand dermatoses in food handlers [64,65]. There are various illustrations, one of which is spice factory workers (for example, in a Swedish spice factory [66]) because their work requires significant physical contact with the ground product of spices [52]. The most common dermatological reaction to spices and their essential oils is reported to be irritant contact dermatitis, although allergic contact dermatitis can also ensue [52]. Spices that are common causative agents include clove, cinnamon, nutmeg, ginger, garlic, allspice (Jamaica pepper), and paprika [67,68]. Certain of these spices are noted to be “commonly used in the European kitchen” [67].

### 3.7. Socioeconomic Factors

Socioeconomic factors may affect the prevalence or severity of dermatitis in different ethnic groups (although not necessarily limited to hand eczema). This includes (a) the varying levels of healthcare utilization across different ethnic groups, including in respect of dermatological conditions [69,70], which could be due to inter alia, cultural or language reasons [71], or economic factors, (b) the hygiene hypothesis combined with the impact of household income and education [25], (c) products targeting specific ethnic populations that may result in different exposure to chemicals based on ethnicity [72,73], and (d) different living conditions resulting in environmental risk. In a Bradford study, children from the “Other” ethnic groups (i.e., ethnicities not of “White British” or Pakistani” origin, which in their study included children from Arab, Chinese, Filipino, and other Asian backgrounds) were noted to be “slightly more likely to have visible damp in their homes” [74]. Furthermore, mold exposure may be higher in children from minority groups (although other environmental exposures may have to be further examined) [75].

### 3.8. Different Laws and Regulations

Another factor could be the varying regulatory regimes to which different ethnic groups may be subject due to their geographical location, which may affect their exposure risk. For example, the European Union (EU) introduced legislation to control nickel content and release from certain consumer items through the EU Nickel Directive 1994, which came into full force in 2001 and is now part of the REACH regulation of the EU. Nickel allergy is widely considered a risk factor for hand eczema [76,77]. In a study of a multi-racial Asian population, no racial predilection to nickel dermatitis was established [78]. Thus, the differing rates of hand eczema post-implementation of nickel-related regulations suggest that the distinct regulatory frameworks may result in increased risk for those with no or relatively less regulatory protection.

## 4. Discussion

### 4.1. Summary

Based on the available literature to date, factors that may account for the disparity in risk of hand eczema in different ethnicities include (a) genes, (b) differing skin physiology, (c) cultural and other related practices, (d) dietary habits and associated food preparation, (e) climate, (f) predominant occupations in certain ethnic groups, (g) socioeconomic factors, and (h) dissimilar laws and regulations. The environmental or lifestyle factors detailed could be non-exhaustive because there is a wide possibility of potential exposures. More definitive evidence may be required in relation to some of these factors.

### 4.2. Conclusions

Given that endogenous risk factors cannot be avoided, but certain exogenous aspects can be modified, it could be helpful from a practical perspective to focus more on addressing the modifiable factors. This is also because there is literature noting that the “frequency of hand dermatitis, flare-ups in particular, is predominantly determined by environmental factors” [11]; hence, the environment could play an important role in causing or worsening hand eczema. Of the aforementioned elements that may impact the prevalence and severity of hand eczema in different ethnicities, the relatively modifiable risk factors pertain to cultural practices, food preparation methods, and certain other customs of various ethnic groups.

Therefore, it would be beneficial for healthcare professionals to be well-acquainted with such factors to be better equipped to tailor the treatment approach for a patient accordingly. This would include taking note of any relevant risk factors during history taking, physical examination, and the conduct of investigations, such as patch testing. Moreover, as the cultural practices of different ethnic groups should be respected, and patients may have to continue working in certain occupations to sustain their livelihood, it may not be feasible to avoid certain activities in entirety. Thus, a healthcare professional could consider whether there are any suitable preventive and/or protective measures (for example, the use of appropriate gloves and moisturizers) that could reduce the impact of environmental exposure. Given how socioeconomic factors can play a role in the viability of treatment, the solutions (for example, an effective emollient) should ideally be affordable for the at-risk groups.

Patient education on adequate measures to protect against hand eczema or reduce exacerbation of such a condition could also be helpful in providing holistic treatments. Patient awareness is vital in particular because some cultural customs take place on a frequent or periodic basis rather than a one-time occurrence.

Furthermore, while the use of protective measures (for example, the use of suitable gloves) can be helpful, these may not resolve the root of the problem. Occupational risks, socioeconomic reasons, and the presence and/or enforcement of laws and regulations can be pertinent determinants in the risk of developing or worsening hand eczema. Hence, policy-related factors should not be disregarded.

In conclusion, the importance for healthcare professionals to be familiar with the risk factors of hand eczema that arise in different ethnic groups may be all the more crucial in view of increasing globalization and cross-border migration. This is because there may be a more diverse patient population, such that cultural practices and customs are not always limited to particular geographic regions [39].

### 4.3. Further Research Areas

Regarding the modifiable risk factors, while the existing literature highlighted some examples of cultural practices, as cited in this article, the findings indicate a paucity of research on other environmental exposures (including other types of cultural practices, customs, and dietary habits). More in-depth examination of other potential contributors, how risks can be reduced, and the effectiveness and feasibility of specific preventive or protective measures and treatment methods given possible psychosocial constraints would be beneficial.

With regards to non-modifiable factors, such as genetics or skin physiology, the extent of their clinical significance and how the treatment should be modified accordingly is not fully clear. The existing literature noted the lack of inclusion of diverse ethnic groups in clinical trials relating to dermatology [20,25,79,80,81] and proposed for a wider variety of ethnicities to be included when conducting clinical trials [82,83]. Therefore, more definitive studies may be warranted to determine the clinical significance of aspects such as genetics and skin physiology and whether treatment plans for different ethnicities have to be customized accordingly.

### 4.4. Limitations

Our review has some limitations. To make the review more feasible, we were only able to include the literature published in English and where full text was accessible. In addition, the scope of dermatological conditions is based on each original article, and certain literature reviewed are not limited to solely hand eczema; thus, further in-depth examination in the context of hand eczema only may be preferable. Furthermore, the results are only up to date as of 10 February 2023.

## Data Availability

Not applicable.
